# Super-Minimal Incision Technique in Pediatric Kidney Transplantation: A Paired Kidney Analysis

**DOI:** 10.3389/fped.2022.862552

**Published:** 2022-04-06

**Authors:** Junxiang Wang, Lixiang Zhao, Guiwen Feng, Wenjun Shang

**Affiliations:** The Department of Kidney Transplantation, The First Affiliated Hospital of Zhengzhou University, Zhengzhou, China

**Keywords:** pediatric kidney transplantation, super-minimal incision, conventional kidney transplantation, postoperative complications, cosmetic result

## Abstract

**Background:**

Recently, the demand for minimally invasive techniques in kidney transplantation (MIKT) has increased. However, there is only a limited number of studies on MIKT, especially in pediatric kidney transplants. Hence, we evaluated whether there is a difference between the super-minimal incision technique in pediatric kidney transplantation (SMIPKT) and conventional kidney transplantation (CKT).

**Methods:**

Between December 2018 and November 2021, 34 patients who underwent pediatric kidney transplantation with a follow-up of 1 month were enrolled. A paired kidney analysis was performed to minimize donor variability and bias. The SMIPKT and CKT groups included 17 patients.

**Results:**

There was no difference in baseline clinical characteristics, including age, sex, the donor/ recipient weight ratio (DRWR), choice of dialysis modality, pretransplant dialysis time, BMI, renal artery number, cause of ESRD, DGF, length of the kidney and cold ischemic time, tacrolimus concentration at 3 and 7 days, serum creatinine at 1 month and postoperative complication rate between the SMIPKT and CKT groups (all *P* > 0.05). However, the length of the incision, operation time, intraoperative bleeding, postoperative drainage volume within 24 h and Vancouver scar scale at 1 month were statistically significant (all *P* < 0.05).

**Conclusion:**

Compared with CKT, our results indicated that SMIPKT showed more satisfactory cosmetic results, shorter SMIPKT operating time, and reduced intraoperative bleeding and postoperative drainage volume within 24 h. There were also no statistical differences in postoperative complications. Hence, we suggest that SMIPKT is an appropriate method for pediatric kidney transplantation.

## Introduction

Minimally invasive surgery (MIS) in laparoscopic living donor nephrectomy has become an optimal choice for many transplant centers (1, 2). Compared with open, conventional operations in living donor nephrectomy, the advantages of MIS include reduced tissue trauma and postoperative pain, and pleasing cosmetic results (3, 4). However, in the past few years, there have only been limited reports of minimally invasive techniques for adult kidney transplantation (MIKT) (5, 6), but few publications on pediatric kidney transplantation (PKT). Considering the potential advantages of reducing incision/tissue trauma in immunosuppressed pediatric kidney transplant recipients, a super-minimal incision technique (~4–6 cm) in pediatric kidney transplantation (SMIPKT) was evaluated in our transplant center. Therefore, the aim of the present study was to evaluate whether there is a difference between SMIPKT and conventional kidney transplantation (CKT) and the effectiveness of SMIPKT in pediatric recipients.

## Materials and Methods

### Experimental Design

This was a single-center retrospective study. A paired kidney analysis was performed to minimize donor variability and bias. Thirty-four grafts from 17 pediatric donors after cardiac death were distributed to our transplant center using China's organ distribution system. Pediatric recipients from the same pediatric donor were divided into the SMIPKT and CKT groups, depending on whether the incision was minimal or not. All surgeons have been performing kidney transplantation for over 10 years.

### Inclusion and Exclusion Criteria

The inclusion criteria were <18 years, first kidney transplantation, and no previous surgery at the transplant site.

The exclusion criteria were older than 18 years, more than two kidney grafts, En bloc or dual kidney transplantation, or only one kidney was distributed to our transplant center by China's organ distribution system.

### Surgical Technique

#### SMIPKT

**Figure 1:**
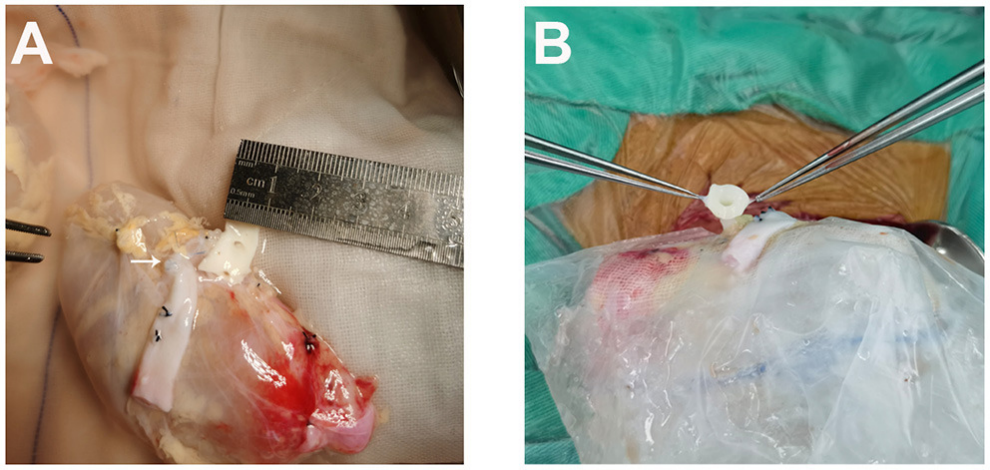
A careful back-table preparation of the kidney. **(A)** The short right renal vein was extended by reconstruction using the vena cava (white arrow). **(B)** Tailored aortic patch.

**Figure 2:**
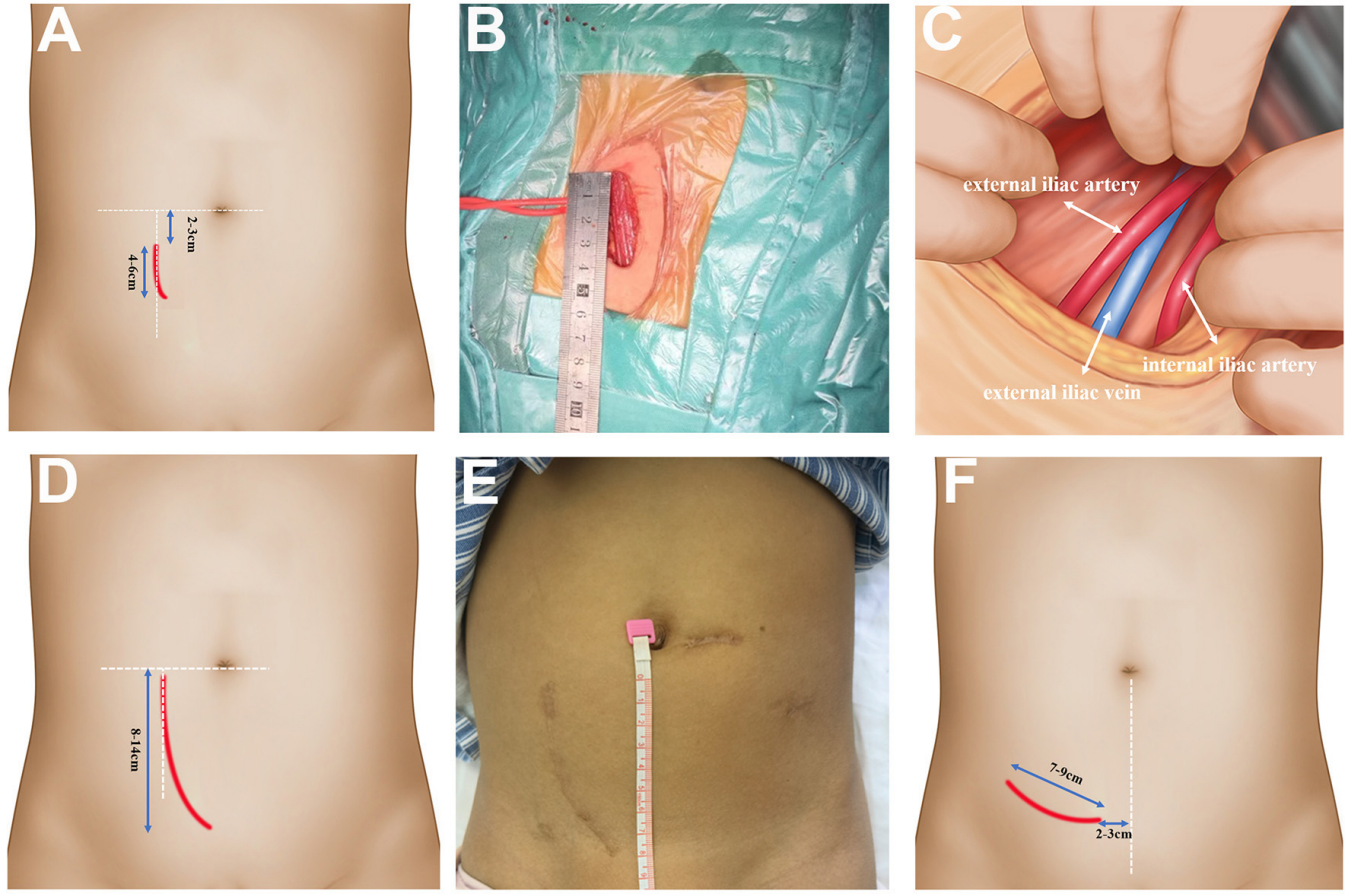
The operative approach of SMIPKT and CKT. **(A)** The operative approach of SMIPKT. **(B)** A ~4 cm incision in SMIPKT in a 9-year-old patient. **(C)** The internal iliac artery and the external iliac vein were fully exposed and dissected free in a minimalistic fashion in SMIPKT. **(D)** The operative approach of CKT. **(E)** A ~8.5 cm incision in CKT (final result 2 years post-transplant in a 9-year-old patient). **(F)** The transverse incision of MIKT by Oyen and Kim.

**Figure 3:**
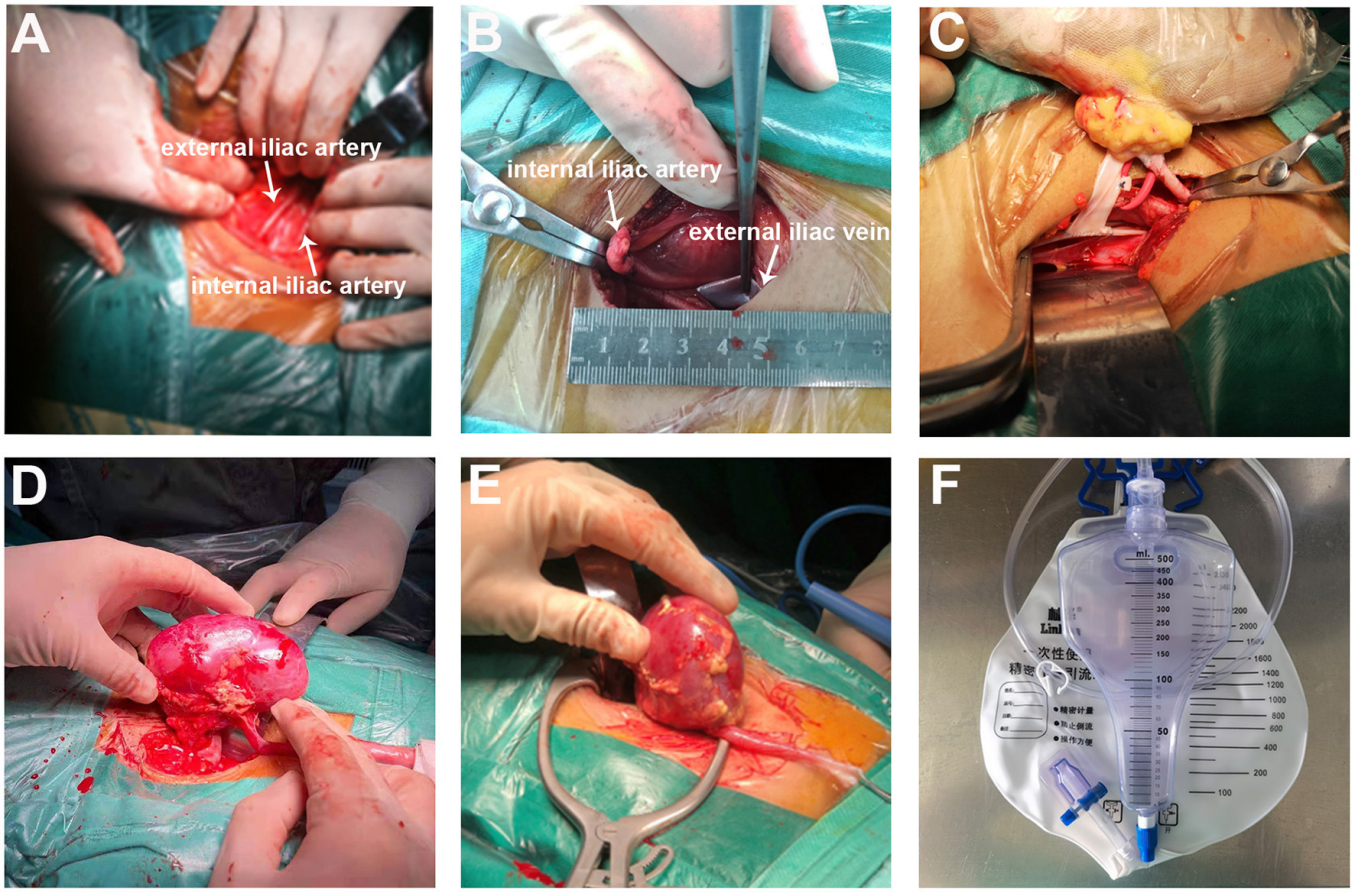
The SMIPKT surgical technique. **(A,B)** Full exposure with minimal dissection of the external iliac artery (white arrow), iliac artery (white arrow), and the external iliac vein (white arrow). **(C)** The two vascular anastomoses were performed with renal graft in a custom-made ice bag wrapped in ice sludge. **(D)** Minimal incision can be smaller than the kidney. **(E)** The upper pole of renal graft was placed into the iliac fossa at first. **(F)** Blood loss and postoperative drainage volume are precisely measured through a self-control precision metering drainage bag.

Before transplantation, we performed a careful back-table kidney preparation (Figures 1A,B). In SMIPKT, we used an incision starting 2–3 cm below the umbilicus and extending 4–6 cm along the outer edge of the rectus abdominis (Figures 2A,B). The length of the incision was selected based on the size of the graft. Only the “conjoined tendon” and hardly any muscular tissue were divided. The inferior abdominal vessels, spermatic cord, or ovoid ligament need not be dissected. The origin of the internal iliac artery and its terminal branches, the external iliac vein, and the bladder can be fully exposed and dissected in a minimalistic fashion (Figures 2C, 3A,B). After the back-table preparation of the kidney was completed, the renal graft was wrapped with gauze, leaving only vessels for anastomosis, and then placed into a custom-made ice bag wrapped with ice sludge for cooling. The graft vein was anastomosed to the external iliac vein (end-to-side), and the graft artery was anastomosed to the internal iliac artery (end-to-end). A continuous suture with 6-0 SurgiPro was used for both vascular anastomoses (Figure 3C). Following anastomosis, intraluminal air was thoroughly excluded by infusing heparin saline. The graft blood flow was then opened (Figures 3D,E). The graft ureter was anastomosed to the recipient's bladder with running sutures using 5-0 absorbable suture via the Lich-Gregoir technique with a 3–4.7 Fr double J stent. All incisions were sutured subcutaneously using 3-0 absorbable sutures. The blood loss of all patients was collected using a negative pressure suction device during surgery. Blood loss and abdominal fluid drainage were precisely measured using a self-control precision metering drainage bag (Figure 3F). The short right renal vein was extended by reconstruction using the vena cava.

#### CKT

Conventional pediatric Gibson's technique has been practiced for years (from 2008 to 2021, 60–100 pediatric kidney transplantations per year) in our transplantation center, with an incision ~8–14 cm in length in the lower right abdomen (Figures 2D,E). The inferior abdominal vessels and ovoid ligament were ligated, and the spermatic cord was dissociated. Through the skin and muscular layers, with wide extraperitoneal exposure of the internal/external iliac artery and external iliac vein, the lymphatic trunks alongside the vessels and the small branches of peripheral blood vessels were ligated. The remaining surgical steps were applied in a similar manner to the patients in the SMIPKT group.

### Postoperative Management

All postoperative patients were admitted to the ICU of the kidney transplantation ward for 5 days. Central venous cannulation, with catheter placement by either the subclavian or internal jugular vein, has been well-established to monitor central venous pressure. Continuous non-invasive blood pressure monitoring is necessary during the 1st week after surgery, and the blood pressure is allowed to fluctuate between 100/60 and 120/80 mmHg. The drainage fluid and urine output were recorded per hour.

### Immunosuppression Protocol

All recipients received a reduced dose of anti-human T lymphocyte rabbit immunoglobulin + methylprednisolone (cumulative 18–21 mg/kg) for preoperative induction and postoperative prevention of rejection. A triple immunosuppressive regimen of CNI combined with mycophenolate mofetil (MMF) and prednisone was administered postoperatively. The initial dose of tacrolimus was determined according to the CYP3A5 genotype, and the target tacrolimus trough concentrations were monitored weekly during the first 3 months.

### Statistical Analysis

The data were analyzed using SPSS for Windows (SPSS, version 19; IBM Corporation, Armonk, NY, USA). Parametric and non-parametric data are presented as mean ± standard deviation (SD) or median with range. Categorical variables are expressed as frequencies and percentages. We used the *t*-test to compare the parametric continuous variables. Meanwhile, the Mann-Whitney *U*-test was used for non-parametric continuous variables. Statistical significance was set at *p* < 0.05.

## Results

The median age was 13 years in the SMIPKT group and 14 in the CKT group, and 64.7% of the patients were male. Among the SMIPKT and CKT groups, other clinical characteristics, including DRWR, the choice of dialysis modality, pretransplant dialysis time, body mass index (BMI), renal artery number, cause of ESRD, DGF, length of the kidney, and cold ischemic time were statistically insignificant (all *P* > 0.05) (Table 1). More details are shown in Table 2.

**Table 1:** Characteristics of patients.

**Variable**	**SMIPKT group**	**CKT group**	***P*-value**
	**(*n* = 17)**	**(*n* = 17)**	
Age (y), median (IQR)	13.0 (6.5–14.5)	14.0 (10.0–15.0)	0.198
Men, *n* (%)	11 (64.7)	11 (64.7)	1.000
DRWR	1.07 (0.54–1.43)	0.93 (0.45–1.14)	0.234
Dialysis, *n* (%)			0.814
HD	6 (35.3)	7 (41.2)	
PD	9 (52.9)	9 (52.9)	
Preemptive	2 (11.8)	1 (5.9)	
Pretransplant dialysis time (months), median (IQR)	9.0 (6.0–24.0)	12.5 (8.5–17.5)	0.342
BMI (kg/m^2^), median (IQR)	15.8 (14.5–17.5)	16.7 (15.0–17.9)	0.428
Multiple renal arteries (≥2), *n* (%)			0.368
2	2 (11.8)	2 (11.8)	
3	0 (0.0)	1 (5.9)	
4	1 (5.9)	0 (0.0)	
Cause of ESRD, *n* (%)			0.765
Primary glomerular diseases	9 (52.9)	8 (47.1)	
Secondary glomerular disease	6 (35.3)	5 (29.4)	
Hereditary nephropathy	1 (5.9)	1 (5.9)	
No data	1 (5.9)	3 (17.6)	
DGF, *n* (%)	0 (0.0)	0 (0.0)	-
Cold ischemic time (h), mean ± SD	12.5 ± 3.0	12.7 ± 2.6	0.913
Length of the kidney (cm), mean ± SD	6.4 ± 1.0	6.3 ± 1.1	0.861
Wound infections	0 (0.0)	0 (0.0)	-
Wound dehiscence	0 (0.0)	0 (0.0)	-
Incisional hernia	0 (0.0)	0 (0.0)	-
Lymphocele	0 (0.0)	0 (0.0)	-
Urologic complications	1 (5.9)	1 (5.9)	1.000
Vascular complications	1 (0.0)	1 (0.0)	1.000
Length of the incision (cm), median (IQR)	4.5 (4.0–5.0)	12 (9.5–13.5)	<0.001
Operation time (min), mean (IQR)	130 (116–143)	162 (136–190)	0.014
Intraoperative bleeding (ml), median (IQR)	40 (30–50)	60 (35–175)	<0.001
Postoperative drainage volume within 24 h (ml), median (IQR)	60 (35–175)	160 (93–360)	0.007
Vancouver scar scale at 1M, median (IQR)	6 (5–6)	7 (6–8)	<0.001
Tacrolimus concentration (ng/ml)at 3d, mean ± SD	13.0 ± 5.0	10.8 ± 3.0	0.137
Tacrolimus concentration (ng/ml)at 1w, mean ± SD	11.1 ± 2.5	10.0 ± 1.7	0.122
Serum creatinine (μmol/L) at 1M, median (IQR)	82 (54–103)	94 (69–124)	0.215

*SMIPKT, super-minimal incision technique in pediatric kidney transplantation; CKT, conventional kidney transplantation; DGF, delay graft function; DRWR, donor/ recipient weight ratio*.

*“-”: the two groups were not compared*.

**Table 2:** Detailed clinic characteristics of patients.

	**Sex**		**Age (years)**		**Weight (kg)**		**BMI (kg/m** ^**2**^ **)**	
***n***	**SMIPKT group**	**CKT group**	**SMIPKT group**	**CKT group**	**SMIPKT group**	**CKT group**	**SMIPKT group**	**CKT group**
1	Male	Female	14	15	35.0	45.0	15.7	17.8
2	Male	Female	11	14	30.0	47.0	14.8	17.7
3	Male	Male	9	15	32.0	41.0	15.8	21.2
4	Male	Male	17	14	53.0	40.0	17.5	16.6
5	Female	Male	6	16	19.0	47.0	17.2	16.2
6	Male	Female	13	5	41.0	12.0	18.7	10.9
7	Male	Male	16	15	38.0	46.0	14.5	16.7
8	Male	Male	15	15	40.0	55.0	16.6	22.0
9	Female	Male	14	14	24.0	27.0	14.2	15.9
10	Female	Female	4	9	14.0	20.0	14.3	14.9
11	Female	Male	8	13	25.0	43.0	17.4	17.9
12	Male	Male	16	14	49.0	34.0	18.4	18.7
13	Female	Female	13	9	51.6	24.4	21.8	14.0
14	Female	Female	14	17	40.0	39.0	16.0	17.3
15	Male	Male	2	11	10.5	24.0	14.5	14.2
16	Male	Male	7	9	20.0	21.0	14.4	15.1
17	Male	Male	4	14	14.0	41.0	14.9	17.1

Naturally, the SMIPKT skin incision was much shorter, and there were significant differences in terms of operative time, intraoperative bleeding, postoperative drainage volume within 24 h, and Vancouver scar scale at 1 month (all *P* < 0.05). Considering the effect of immunosuppressants on wound healing, tacrolimus trough concentration was monitored on postoperative days 3 and 7, and there was no significant difference between the SMIPKT and CKT groups (*P* > 0.05). Although the serum creatinine level at 1 month in the SMIPKT group was slightly lower than that in the CKT group, there was no statistically significant difference between the two groups (*P* > 0.05) (Table 1).

During the 30 days follow-up, there was no wound infection, wound dehiscence, incisional hernia, or lymphocele. However, one pediatric recipient with a diagnosis of urologic stenosis in the SMIPKT group and one with a diagnosis of urine leakage in the CKT group was confirmed by computerized tomography (CT) urography. A child with urologic stenosis underwent early ureteral reimplantation. Two pediatric recipients with stable graft renal function and no hypertension (1 in each group, *P* = 1.000) had a confirmed vascular complication of transplant renal artery stenosis on CT angiography.

The primary diseases of all patients are shown in Table 1. However, two patients showed non-surgical-related complications. One patient in each group relapsed with focal segmental glomerular sclerosis (FSGS) in the early postoperative period after kidney transplantation.

## Discussion

A major point of the present MIS is the reduction of tissue trauma by using limited-sized incisions to obtain less pain and scarring, and a faster recovery period compared to traditional surgery (7, 8). Surprisingly, the original open technique of an oblique Gibson incision of ~20 cm in length remains the gold standard since kidney transplantation was first successfully performed in the 1950's.

MIKT, using a 7–9 cm transverse incision, 3–5 cm above the inguinal ligament, was first described in 21 kidney transplants by Oyen in 2006 (6). Kim (5), Kacar (9), and other similar series published later confirmed that MIKT is feasible and safe (10). Although there are few publications about MIKT in adults, we believe that SMIPKT is feasible and a more obvious choice for several reasons. First, pediatric recipients with end-stage renal disease often experience anemia, hypoalbuminemia, edema, chronic malnutrition, and growth retardation. Furthermore, immunosuppressive drugs must be taken daily or twice daily; in particular, steroids and mycophenolate acid significantly impair wound healing ability. Therefore, pediatric kidney transplant recipients may have a higher risk of wound infection, poor wound healing, and other wound complications than non-immunosuppressed patients after open surgery. Second, some children, especially younger ones, have poor compliance. The advantages of SMIPKT include reduced tissue trauma and pain, faster recovery, and better compliance. Third, the long-term mental health of children after kidney transplantation is important, and small incisions with cosmetic results can weaken the impact of kidney transplants. Fourth, the majority of pediatric candidates in our transplantation center were underweight, with a low BMI ( ≤ 20 kg/m^2^) due to chronic malnutrition. Moreover, the surgical field could be adequately exposed and clearly visible. Fifth, all renal grafts from pediatric donors after cardiac death were small for size, with an average diameter of 6.4 ± 1.0 cm in the SMIPKT group and 6.3 ± 1.1 cm in the CKT group, which did not require a large surgical space. Thus, a smaller incision approach could be easier to achieve.

In our study, among the baseline clinical characteristics, age, sex, DRWR, choice of dialysis modality, pretransplant dialysis time, BMI, renal artery number, cause of ESRD, DGF, length of the kidney and cold ischemic time, tacrolimus concentration at days 3 and 7, serum creatinine at 1 month were found to be statistically insignificant. However, the length of the incision, operation time, intraoperative bleeding, postoperative drainage volume within 24 h, and Vancouver scar scale at 1 month were statistically significant between the SMIPKT and CKT groups. We thought that SMIPKT may cause a higher complication rate in vascular and urinary complications, but there were no statistically significant differences in urologic complications (i.e., urologic stenosis or urine leakage), vascular complications (anastomotic stenosis), lymphocele, wound dehiscence, wound infections, and incisional hernia between SMIPKT and CKT groups, which is similar to other reports (6, 9).

Although there was no statistically significant difference in BMI between the SMIPKT and CKT groups, whether BMI really affects SMIPKT is unclear. Despite some limitations in space in obese patients using the small incision technique, there is no drawback in performing the anastomoses in overweight patients up to a BMI of 30 kg/m^2^ in Claas Brockschmidt's and >30 kg/m^2^ in Oyen's report (4, 6). However, in a subsequent study by Kim, a BMI <25 kg/m^2^ was the inclusion criterion for MIKT recipients to ensure adequate exposure to the surgical field (5). Although SMIPKT has been proven successful in pediatric candidates with a BMI >25 kg/m^2^ in our transplantation center, it should be noted that the majority of pediatric candidates in our group were lean, with a BMI of <20 kg/m^2^, which was much lower than the previously described results. This may be a beneficial factor for SMIPKT. However, considering the limitations in surgical space in obese patients, candidates with a BMI ≥ 30 are still recommended for traditional kidney transplantation technique.

It has not been established whether DRWR mismatch truly affects the application of the super-minimal incision technique. According to our clinical experience with pediatric kidney transplantation, a DRWR mismatch ≤ 3.0 (maximum in this study) is not critical to preventing the use of the super-minimal incision technique. The width of the transplanted kidney (not kidney length) and the length of the recipient internal iliac artery that is sufficient to anastomose the graft artery (end to end) are the two key factors for the successful use of the super-minimal incision technique. However, considering the DRWR > 3, we prefer to recommend the CKT technique.

The operative approach is the key to SMIPKT. Oyen and Kim made a 7–9 cm transverse incision (Figure 2F). It is located 3–5 cm above the groin, with the medial endpoint of the incision 2–3 cm from the midline (5, 6). The difference was that the graft artery was anastomosed to the internal iliac artery at our transplant center. Hence, we used an incision starting 2–3 cm below the umbilicus and extending 4–6 cm along the outer edge of the rectus abdominis in SMIPKT. Only the “conjoined tendon” and hardly any muscular tissue were divided. The origin of the internal iliac artery and its terminal branch, the external iliac vein, and the bladder can be fully exposed and dissected in a minimalistic fashion. Currently, the minimum age and weight of the pediatric recipients using the super-minimal incision technique at our transplant center are 1 year old and 5 kg. However, in very young recipients with extremely low body weight ≤ 5 kg or <1 year old, the graft artery is often anastomosed with the common iliac artery or the distal aorta (end-to-side). The super-minimal incision does not provide sufficient surgical space for easy dissection of the distal aorta and vascular anastomosis, as well as vesicoureteral replantation using the Lich-Gregoir technique. Therefore, the traditional pediatric kidney transplantation is recommended for very young recipients with extremely low body weight ≤ 5 kg or <1 year old. Moreover, venous anastomosis sites should be evaluated before transplant surgery. A suitable venous anastomosis site and well-tailored allograft vein can avoid kinking. Meanwhile, after reperfusion of the transplanted kidney, the final position of the transplanted kidney can also be adjusted (including adjusting the angle of the allograft or allograft was implanted higher in the retroperitoneal iliac fossa) to avoid kinking.

Another criticism of SMIPKT is that it does not sufficiently cool renal grafts before revascularization. To avoid this, the kidney space was pre-cooled with ice sludge for 5 min in advance. The kidney was then placed with the retroperitoneal pouch into the iliac fossa, and all three anastomoses were performed in the final position. It is not possible to move the kidney from a nearly fitted retroperitoneal pouch. Compared to CKT, the duration of MIKT may be prolonged for all three anastomoses. Studies have revealed that prolonged anastomosis time leads to significantly inferior long-term graft outcomes and patient survival (11, 12). Furthermore, if the renal hilum is bleeding after reperfusion, the kidney needs to be removed from the incision for hemostasis and must be reimplanted into the iliac fossa. When the original incision is insufficient, it must be extended. Therefore, we prefer to perform the two vascular anastomoses outside of the retroperitoneal cavity with renal graft in a custom-made ice bag wrapped in ice sludge, leaving more space, better vision, a simpler inspection of the kidney, and hemostasis after reperfusion.

Our results showed that SMIPKT has a shorter operating time and more favorable cosmetic effects. Moreover, its use seems particularly promising among the immunosuppressed population. Based on our positive experience with SMIPKT, we found it to be technically feasible, and it can be executed safely and quickly by any experienced kidney transplant surgeon after a very short learning curve. Considering that SMIPKT is not only suitable for pediatric recipients with the end-stage renal disease but can also weaken the impact of kidney transplants in children's growth (especially in younger children), we believe that SMIPKT is worth recommending.

The shortcomings of the experimental design are as follows. First, the research took place at a single center with a small number of cases, which needs to be further supplemented. Second, the patients were followed up for a short time; hence, long-term follow-up is still needed.

## Data Availability Statement

The original contributions presented in the study are included in the article/supplementary material, further inquiries can be directed to the corresponding author.

## Ethics Statement

The studies involving human participants were reviewed and approved by the First Affiliated Hospital of Zhengzhou University Ethics Committee. Written informed consent for participation was not required for this study in accordance with the national legislation and the institutional requirements.

## Author Contributions

WS: conceived, designed, and supervised the study. WS, JW, and LZ: performed the experiments. JW: performed data analysis and wrote the paper. All authors revised and reviewed the paper. All authors contributed to the article and approved the submitted version.

## Funding

This study was supported by Youth Fund of the First Affiliated Hospital of Zhengzhou University (YNQN2017097).

## Conflict of Interest

The authors declare that the research was conducted in the absence of any commercial or financial relationships that could be construed as a potential conflict of interest.

## Publisher's Note

All claims expressed in this article are solely those of the authors and do not necessarily represent those of their affiliated organizations, or those of the publisher, the editors and the reviewers. Any product that may be evaluated in this article, or claim that may be made by its manufacturer, is not guaranteed or endorsed by the publisher.
